# Adenosine A_1_ receptor activation mediates the developmental shift at layer 5 pyramidal cell synapses and is a determinant of mature synaptic strength

**DOI:** 10.1113/jphysiol.2012.244392

**Published:** 2013-04-22

**Authors:** Michael I Kerr, Mark J Wall, Magnus J E Richardson

**Affiliations:** 1 Warwick Systems Biology Centre, University of Warwick Coventry, UK; 2 School of Life Sciences, University of Warwick Coventry, UK; 3 Warwick Systems Biology Doctoral Training Centre, University of Warwick Coventry, UK

## Abstract

During the first postnatal month glutamatergic synapses between layer 5 pyramidal cells in the rodent neocortex switch from an immature state exhibiting a high probability of neurotransmitter release, large unitary amplitude and synaptic depression to a mature state with decreased probability of release, smaller unitary amplitude and synaptic facilitation. Using paired recordings, we demonstrate that the developmental shift in release probability at synapses between rat somatosensory layer 5 thick-tufted pyramidal cells is mediated by a higher and more heterogeneous activation of presynaptic adenosine A_1_ receptors. Immature synapses under control conditions exhibited distributions of coefficient of variation, failure rate and release probability that were almost coincident with the A_1_ receptor blocked condition; however, mature synapses under control conditions exhibited much broader distributions that spanned those of both the A_1_ receptor agonized and antagonized conditions. Immature and mature synapses expressed A_1_ receptors with no observable difference in functional efficacy and therefore the heterogeneous A_1_ receptor activation seen in the mature neocortex appears due to increased adenosine concentrations that vary between synapses. Given the central role demonstrated for A_1_ receptor activation in determining synaptic amplitude and the statistics of transmission between mature layer 5 pyramidal cells, the emplacement of adenosine sources and sinks near the synaptic terminal could constitute a novel form of long-term synaptic plasticity.

**Key points**

- Neocortical layer 5 pyramidal cell synapses exhibit a developmental reduction in neurotransmitter release probability. Mature synapses are weaker, less reliable and show greater facilitation than immature connections.
- Using paired intracellular recordings our study identifies the mechanism that mediates this developmental change as being due to an increased activation of presynaptic adenosine A_1_ receptors.
- Unlike immature connections, which showed little A_1_ receptor activation, mature connections demonstrated a broad range of activation that was inversely correlated to mature synaptic strength.
- We show that the functional efficacy of A_1_ receptors does not change over development and so our evidence points to concentrations of extracellular adenosine at synapses increasing locally over development.
- The increased adenosine levels significantly affect synaptic efficacy suggesting that the emplacement of adenosine sources and sinks might be a novel mechanism for long-term plasticity at layer 5 pyramidal cell synapses.

## Introduction

Many cortical glutamatergic synapses exhibit a developmental reduction in the probability of neurotransmitter release (Feldmeyer & Radnikow, 2009). This developmental shift has been studied in the major excitatory connections in the somatosensory (Reyes & Sakmann, 1999; Frick *et al.* 2007), auditory (Oswald & Reyes, 2008), visual (Cheetham & Fox, 2010; Etherington & Williams, 2011) and prefrontal (Gonzalez-Burgos *et al.* 2008) cortices and typically occurs between the second and fourth postnatal week in rodents. Synapses between large, thick-tufted layer 5 pyramidal cells (L5PCs) are representative with unitary EPSP amplitudes reduced from 1.3 mV (Markram *et al.* 1997) to 0.3 mV (Reyes & Sakmann, 1999) and short-term plasticity changing from depression to facilitation. This change coincides with the period of critical plasticity in the rodent, with the onset of hearing and eye opening around the end of the second postnatal week, and contributes to the sharpened response of the neocortical microcircuit to sensory stimuli (Oswald & Reyes, 2008; Etherington & Williams, 2011).

Over the same period these excitatory connections increase in variability and decrease in reliability (Frick *et al.* 2007; Etherington & Williams, 2011) suggesting a presynaptic locus for the developmental shift. Previous investigations provided evidence that the increased variability arises from a reduction in the probability of neurotransmitter release, in some cases by greater than half (Iwasaki & Tomoyuki, 2001). The maturation of these central excitatory synapses has therefore been linked to processes that reduce transient calcium concentrations in the presynaptic terminal (Feldmeyer & Radnikow, 2009) but to our knowledge the specific mechanism has not been identified.

One neuromodulator affecting excitatory neurotransmitter release is the purine adenosine. Adenosine is released during physiological and pathological activity (Fredholm, 1996) but significant levels of endogenous adenosine have also been reported in quiescent tissue. The adenosine A_1_ receptor (A1R) subtype is the most prevalent in the neocortex (Dunwiddie & Masino, 2001) with expression on both pyramidal cell bodies and axons (Rivkees *et al.* 1995). Activation of presynaptic A1Rs at excitatory synapses by bath-applied adenosine inhibits glutamate release by reducing calcium influx (Dunwiddie & Masino, 2001) decreasing hippocampal and neocortical EPSPs by ∼80% (Dunwiddie & Haas, 1985; Varela *et al.* 1997), increasing variability in transmission and changing synaptic dynamics from depression to facilitation.

An increased activation of presynaptic A1Rs is therefore a promising candidate mechanism for the developmental switch. To investigate this hypothesis and the role A1Rs might play in determining synaptic amplitude, we examined transmission between L5PCs in the immature (around the time of weaning, postnatal days P17–P22) and mature (1–2 weeks older, P27–P32, young adult) rat somatosensory cortex under control conditions, A1R block, or bath-applied adenosine to estimate the level and heterogeneity of A1R activation by endogenous adenosine at different developmental stages.

## Methods

### Slice preparation

Male Wistar rats, P17–P32, were killed by cervical dislocation and decapitated in accordance with the UK Animals (Scientific Procedures) Act 1986. Rats were kept on a 12 h light–dark cycle with slices made 90 min after entering the light cycle. The brain was rapidly removed, cut down the midline and the two sides stuck down. Parasagittal neocortical slices were cut at an angle of 20 deg (to ensure integrity of apical dendrites) with a Microm HM 650V microslicer in cold (2–4°C) high Mg^2+^, low Ca^2+^ artificial cerebrospinal fluid (aCSF), composed of (mm): 127 NaCl, 1.9 KCl, 8 MgCl_2_, 0.5 CaCl_2_, 1.3 KH_2_PO_4_, 26 NaHCO_3_, 10 d-glucose, pH 7.4 when bubbled with 95% O_2_ and 5% CO_2_, 300 mosmol l^−1^). Slices were stored in aCSF (1 mm MgCl_2_, 2 mm CaCl_2_) at 34°C for 30 min then at room temperature for 1–6 h before recording.

### Electrophysiology

Slices were transferred to the recording chamber and perfused at a constant flow rate of 2.5 ml min^−1^ with aCSF at 32 ± 0.5°C. All tubing was gas tight (Tygon) and solutions were strongly bubbled to prevent hypoxia. The slice was visualized using IR-DIC optics with an Olympus BX51W1 microscope and Hitachi CCD camera (Scientifica, Bedford, UK). Whole-cell recordings were made from 2–4 neighbouring thick-tufted layer 5 pyramidal neurons in somatosensory cortex (hindlimb) using patch pipettes (3–8 MΩ) manufactured from thick-walled borosilicate glass (Harvard Apparatus, Edenbridge, UK) and containing (mm): potassium gluconate 135, NaCl 7, Hepes 10, EGTA 0.5, phosphocreatine 10, MgATP 2, NaGTP 0.3 and biocytin 1 mg ml^−1^ (290 mosmol l^−1^, pH 7.2). Thick-tufted layer 5 pyramidal cells were identified by their position in the slice, characteristic current–voltage relationship and morphology. Voltage recordings were obtained using Axon Multiclamp 700B amplifiers (Molecular Devices, USA) and digitized at 20 kHz (Axon Digidata 1440a). Data acquisition was performed using pCLAMP 10 (Molecular Devices). If synaptic connectivity was detected between pairs of neurons then trains of action potentials (8–10) at 15–50 Hz were elicited by 5 ms current pulses in the presynaptic neuron. Unitary EPSP amplitudes were monitored for at least 5 min before recording. Stimulus trains were separated by 10 s and sweeps repeated 30–100 times.

### Drugs and histology

All drugs were prepared as concentrated stock solutions (10–100 mm), stored frozen and then thawed and diluted in aCSF immediately before use. Adenosine, 8-cyclopentyl-theophylline (8CPT; A1R antagonist) and *N*^6^-cyclopentyladenosine (CPA; A1R agonist) were purchased from Sigma (Poole, UK). Adenosine acts via A_1_, A_2_ and A_3_ receptors, A1Rs being the principal neocortical subtype (Dunwiddie & Masino, 2001). Initial experiments confirmed this by comparing transmission in 8CPT + adenosine and 8CPT only, where any difference would be due to A_2_ or A_3_ activation. No significant difference was found, with a mean ratio of 8CPT + adenosine/8CPT of 0.93 (SEM, 0.08). Following recordings slices were processed for identification.

### Data analysis

**Figure 1:**
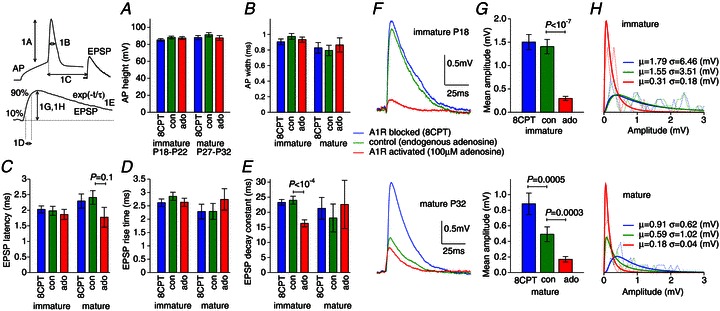
Effect of endogenous adenosine on L5PC synaptic transmission *A*, AP amplitude; *B*, AP width; *C*, AP EPSP latency; *D*, EPSP rise time; *E*, EPSP decay constant: showed no significant change under conditions of A1R block (8CPT, blue), endogenous A1R activation in control (green) or strong A1R activation (applied adenosine, red) within a developmental stage, with the exception of the immature decay constant in adenosine (ado). However, the sweep-averaged EPSP amplitude was significantly affected by A1Rs. *F*, example sweep-averaged EPSP waveforms for immature (P18) and mature (P32) connections. The immature A1R block (blue) and control EPSPs have similar amplitudes whereas it is the mature A1R activated (red) and control EPSPs that are more similar. *G*, mean sweep-averaged amplitudes in A1R block, control and A1R activation show little endogenous A1R activation for immature connections but significant levels of A1R activation for mature connections. The near-identical ratios between A1R block and strong A1R activation for both immature and mature connections demonstrate an unchanged functional efficacy of A1Rs. *H*, log–normal fits to the EPSP distributions for each condition.

**Figure 2:**
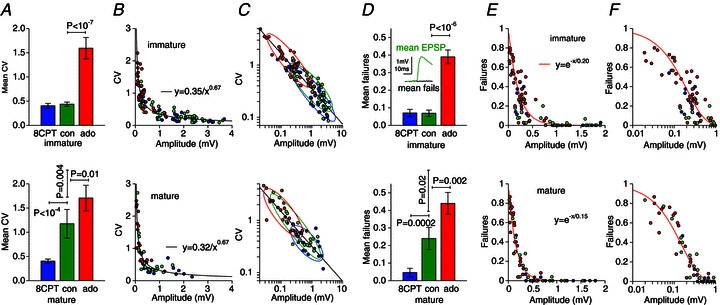
A1R activation determines variability and reliability at mature L5PC synapses through reduced release probability *A*, mean coefficients of variation (CVs) for immature (upper panel) and mature (lower panel) EPSPs for the three pharmacological conditions: A1R block (8CPT, blue), control (green) or A1R activation (bath-applied adenosine, red). The mature control CV is significantly larger than the immature CV but reverts to its immature value on A1R block. The similar ratios of the A1R-blocked (blue) to A1R-activated (red) conditions at the two developmental stages demonstrate unchanged A1R functional efficacy; hence, it is endogenous adenosine concentrations that increase over development. *B*, scatter plots of CV *versus* average amplitude. Data from different pharmacological conditions follow a similar empirical power law for immature and mature connections. *C*, log–log plots of data in *B*. Bivariate Gaussian fits give an indication of the scatter of data under different pharmacological conditions. Control and A1R-blocked distributions coincide for immature connections whereas for mature connections the control distribution exhibits much greater spread overlapping with the A1R antagonized and agonized conditions. *D*, mean failure rates showing the same pattern as the CVs. Inset demonstrates validity of failure criterion. *E*, failure *versus* amplitude; *F*, log amplitude showing the mature control failure rates overlapping with the A1R-blocked and A1R-activated conditions. Exponential fits to the A1R-activated conditions give mean quantal amplitude estimates of 0.20 mV (immature) and 0.15 mV (mature) in adenosine.

Data were analysed using custom MATLAB scripts (MathWorks Inc., Natick, MA, USA). Single sweep and mean unitary EPSPs were measured from baseline (from a 1–3 ms window average before EPSP onset) to peak (with a 0.5 ms window average at EPSP maximum). Overlapping EPSPs were measured using the voltage deconvolution method (Richardson & Silberberg, 2008), failures were measured as in Markram *et al.* (1997) but if d*V*/d*t* > 0.075 mV ms^−1^ the transmission was not considered a failure (see example in Fig. 2*D* inset), and other quantities were measured as described in Fig. 1. Error bars in figures and numbers in parentheses in text are SEMs. Significance values were calculated using the Mann–Whitney test for unpaired data and the Wilcoxon signed-rank test for paired data. N represents the number of experiments performed.

### Short-term synaptic plasticity model

The depression–facilitation model of Tsodyks *et al.* (1998) was used. A train comprising a number of EPSPs *k*= 1, 2, … is considered where each amplitude may be written in the form of *A_k_*=*A*_0_ *x_k_u_k_* where *A*_0_ (mV) is the maximum possible amplitude, *x_k_* (range 0–1) is the fraction of resources available just before action potential (AP) *k* arrives and *u_k_* (range *p*–1) is the fraction of these resources that are utilized on the arrival of AP *k*. The iterative rules for *x_k_* and *u_k_* are


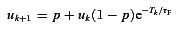
(1)


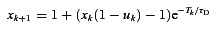
(2)

with initial conditions *x*_1_= 1 and *u*_1_=*p* so that a unitary EPSP (or the first EPSP of a train) would have amplitude *A*_0_*p* where the quantity *p* is the release probability. The interval between pulses *k* and *k*+ 1 is *T_k_*. Together with the maximum amplitude *A*_0_, the parameters of the model are facilitation τ_F_ and depression τ_D_ time constants and utilization parameter *p*. In binomial models of release *x_k_* can be related to the fraction of readily releasable vesicles present before AP *k* and *u_k_* is the probability that a vesicle present is released. The model fit was by lowest mean squared error over an exhaustive parameter search with resolutions: 0.05 mV for *A*_0_, 0.02 for *p*, 20 ms for τ_D_ and 5 ms for τ_F_. It was only necessary to optimize *p* for each pharmacological condition with *A*_0_, τ_D_ and τ_F_ being optimized for all conditions simultaneously, for a particular connection.

### Concentration–response curves

The concentration–response curves for mean unitary EPSP amplitude *E* at bath-applied adenosine concentration *A* were modelled by the logistic form *E*=*E*_min_+ (*E*_max_−*E*_min_)*F*(*A*), where


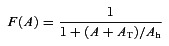
(3)

is the relative amplitude. The parameters of the fit were the minimal *E*_min_ and maximal *E*_max_ EPSP amplitudes, the bath-equivalent endogenous adenosine tone concentration *A*_T_ and the bath-equivalent half-activation *A*_h_. As well as in CPA and 8CPT, EPSP amplitudes were measured in control *A*= 0 μm, *A*= 10, 30 and 100 μm. In order of A1R activation the applied concentrations were equivalent to [−*A*_T_, 0, 10, 30, 100, ∞] where the first and last entries correspond to application of 8CPT and CPA, respectively. The measured amplitudes for these five cases were compared with the predictions from eqn (3) for a trial parameter set {*E*_min_, *E*_max_, *A*_T_, *A*_h_} and the mean squared error between the two were minimized over an exhaustive search with resolutions: *E*_min_, *E*_max_ voltage step 0.02 mV and *A*_T_, *A*_h_ over a logarithmic scale from 0.01 to 100 and 0.1 to 1000, respectively, with 65 intervals. If the best-fit value of *A*_T_ was 0.01 μm it was concluded that no measurable endogenous adenosine was present. The average half-activation concentration 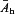 was calculated using a geometric mean, the mean relative amplitudes 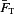 in control (due to the endogenous tone) were calculated as the arithmetic mean of 1/(1 +*A*_T_/*A*_h_) over each of the connections measured. The equivalent mean adenosine tone is then 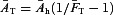. The mean data points (for 0, 10, 100 μm adenosine) were calculated by taking geometric means over the concentrations and arithmetic means of the relative amplitudes.

### Spread of A1R activation within a neocortex

**Figure 4:**
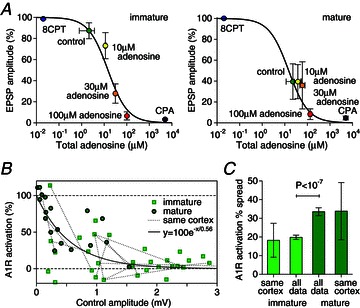
Endogenous adenosine concentrations increase over development and vary significantly between synapses *A*, average adenosine concentration–response curves with relative EPSP amplitude (*E*−*E*_cpa_)/(*E*_8cpt_−*E*_cpa_) *versus* total adenosine concentration (sum of bath-applied and estimated bath-applied-equivalent endogenous adenosine) for immature (*N*= 6) and mature (*N*= 4) connections. Amplitudes in the A1R antagonist 8CPT and agonist CPA were plotted at concentrations 0.05 μm and 500 μm for comparison. *B*, relative A1R activation (*E*_8cpt_−*E*_con_)/(*E*_8cpt_−*E*_ado_) covaries with control amplitude (immature, squares; mature, circles) with an exponential fit capturing the trend. *C*, comparison of median spread across all data and spread in connections from the same neocortex (joined with dotted lines in *B*) for immature and mature synapses. The lack of significant difference between same neocortex data and all data provides evidence that the heterogeneity is at the level of L5PC connections rather than between individuals.

To examine the variability in A1R activation the relative activation *a*= (*E*_8cpt_−*E*_con_)/(*E*_8cpt_−*E*_ado_) was calculated. Let 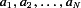 be the set of relative activations measured at a developmental stage and let 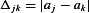 be the absolute difference of two such activations. In total there are *N*(*N*− 1)/2 distinct differences corresponding to all combinations of vertical distances between pairs of points for a given developmental stage in Fig. 4*B*. The median of these *N*(*N*− 1)/2 differences gives a measure of the spread of all the data. To examine variability within a neocortex we now restrict ourselves to connections that were measured in the same slice (linked by dotted lines in Fig. 4*B*). For example, if connections 1, 2 and 5, 6, 7 were measured in two different individuals then the spread for the same-individual data would be the median of 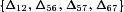. Comparing the spread for all data *versus* that from the same individuals provides a test of the uniformity of A1R activation across synapses in an individual neocortex.

## Results

### A1R activation and synaptic transmission

Using paired recordings, we examined the characteristics of synaptic transmission between L5PCs in the immature (P17–P22) and the mature (P27–P32) somatosensory cortex under various conditions of A1R activation (Fig. 1): A1R block by the antagonist 8CPT (blue); control conditions, to measure A1R activation by endogenous adenosine (green); and strong A1R activation by 100 μm bath-applied adenosine (red). The level of A1R activation had little effect on AP amplitude or width (Fig. 1*A* and *B*) within developmental stages, and the maturation of the control characteristics was in agreement with previous findings (Etherington & Williams, 2011). There was also little effect of A1Rs on the EPSP latency (Fig. 1*C*), rise (Fig. 1*D*) or decay times (Fig. 1*E*) except for the faster EPSP decay for immature connections in adenosine (*P* < 10^−4^; immature *N*= 23, mature *N*= 13 for Fig. 1*A*–*E*). Therefore, adenosine had little effect on the AP shape or initial part of the EPSP waveform.

The amplitude of sweep-averaged EPSPs, however, was significantly affected by the level of A1R activation in both the immature and mature neocortex. A typical immature (P18) connection (Fig. 1*F*, upper panel) showed only a small increase in amplitude above control on A1R block, demonstrating weak A1R activation in control; however, functional A1Rs were present because the same connection reduced by 85% in applied adenosine. Conversely, a mature (P32) connection (Fig. 1*F*, lower panel) showed a small reduction in applied adenosine but a large increase above control on A1R block demonstrating a significant level of A1R activation by endogenous adenosine in control. These characteristics were seen across connections (Fig. 1*G*) with mean sweep-averaged EPSP amplitudes in 8CPT, control and bath-applied adenosine of 1.5(0.2), 1.4(0.2), 0.28(0.05) mV (*N*= 40) in the immature and 0.88(0.1), 0.49(0.1), 0.17(0.04) mV (*N*= 21) in the mature neocortex.

Comparing the data in Fig. 1*G* within developmental stages first, the similarity in mean amplitude of control and A1R-blocked connections implies a weak endogenous activation of A1Rs in the immature neocortex. For mature connections the ratio of mean control amplitude to the A1R-blocked case was 55% demonstrating a significant (*P*= 0.0005) but not maximal endogenous A1R activation. Comparing now between developmental stages, the mean mature control EPSP amplitude dropped to 35% of its immature value, in agreement with previous findings (Reyes & Sakmann, 1999). However, the mature A1R-blocked amplitude dropped by significantly less (*P*= 0.005) to 59% of the immature A1R-blocked amplitude. Hence, the developmental reduction in mean control amplitude is due to increased A1R activation and an A1R-insensitive mechanism in roughly equal proportion. Furthermore, the relative effects of A1Rs, as measured by the ratio of the bath-applied adenosine to A1R-blocked conditions, were indistinguishable at the two developmental stages (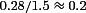 for immature and 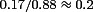 for mature connections). From this observation it can be inferred that the sensitivity of A1Rs is unchanged at immature and mature connections. The amplitude data, together with log–normal fits are provided in Fig. 1*H* for comparison of the distributions.

### A1R activation underlies mature EPSP variability

To investigate the presynaptic and postsynaptic loci of the developmental shift we measured the coefficient of variation (CV) of EPSP amplitudes under the different pharmacological conditions. The mean CVs (Fig. 2*A*) for the 8CPT, control and applied-adenosine conditions were 0.40(0.05), 0.44(0.04) and 1.6(0.2) (*N*= 40) for immature and 0.40(0.04), 1.2(0.3) and 1.7(0.3) (*N*= 21) for mature synapses. The patterns within the two developmental stages were the same as the mean amplitude (Fig. 1*D*): the immature control CV was similar to the A1R-blocked condition (both low CVs) and significantly different (*P* < 10^−7^) to the applied adenosine condition (high CV), whereas the mature control CV was significantly different to both the A1R-blocked (*P* < 10^−4^) and A1R-activated (*P*= 0.01) conditions. Because the number of connections *n* will not change in the short duration between pharmacological manipulations, and the 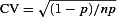 does not depend on quantal amplitude *q*, these results confirm the presynaptic effect (Dunwiddie & Haas, 1985) of A1Rs through reduced probability *p* of neurotransmitter release.

Comparing now between developmental stages, the mean control CVs were significantly different (*P*= 0.004) with mature synapses showing an almost threefold increase consistent with previous studies (Feldmeyer & Radnikow, 2009; Etherington & Williams, 2011). Unlike the mean amplitudes (Fig. 1*D*), the A1R-blocked CVs did not change over development. This suggests that the A1R-independent component of the developmental amplitude reduction is due to a smaller quantal amplitude (as measured from the soma) because *q* is the only binomial quantity that amplitude *E*=*npq* depends on but the CV does not.

We plotted the CV *versus* amplitude for individual immature and mature connections (Fig. 2*B*) for all pharmacological conditions to examine correlations between EPSP variability and amplitude. Synapses under control conditions followed the reciprocal distribution previously reported (Markram *et al.* 1997). Unexpectedly, the connections under A1R antagonized or agonized conditions also fell on the same curve rather than forming distinct clusters. A power law provided a good description of trend with CV = 0.35/*E*^0.67^, where *E* is the average EPSP amplitude. Mature synapses fell on a very similar curve CV = 0.32/*E*^0.67^, although stronger synapses were less abundant as expected. We replotted the data on a log–log scale (Fig. 2*C*) and fitted bivariate Gaussians to give an indication of the distribution for each pharmacological condition. For the immature connections the control and A1R-blocked distributions were nearly coincident and there was little overlap with the distribution for adenosine. For the mature synapses the control distribution comprised connections that overlapped with the domains of both the A1R agonized and antagonized distributions suggesting a diverse range of A1R activation at mature L5PC synapses.

### A1R activation and mature EPSP reliability

To further test the presynaptic effects of A1R activation on the reliability of synaptic transmission, we measured the failure rate for the different conditions (Fig. 2*D*). Under A1R block, control and strong A1R activation the mean failure rate was 0.07(0.02), 0.07(0.02) and 0.39(0.04) (*N*= 40) for immature connections and 0.05(0.02), 0.24(0.06) and 0.44(0.06) (*N*= 21) for mature connections. The pattern of the data was similar to the CV with the higher mature control failure rate reverting to its juvenile value on the blockade of A1Rs: hence, the low reliability in mature synapses is due to activation of A1Rs by endogenous adenosine. The similarity of the A1R-blocked conditions over development provides further evidence that the A1R-independent component of the developmental reduction in EPSP amplitude is due to decreased effective quantal amplitude because the failure rate (1 −*p*)*^n^*, like the CV, is independent of this quantity.

The failure-*versus*-amplitude data scatter (Fig. 2*E* and *F* log amplitude) shows the control distribution spread increasing, from being coincident with the A1R-blocked case for immature connections, to overlapping with the A1R-activated and -blocked cases for mature connections. For small release probability the binomial model predicts an exponential relation between failures and amplitude, with the fit constant equal to the quantal amplitude. Fits (Fig. 2*E* and *F*) to the A1R-activated conditions give quantal amplitude estimates of 0.20 mV for immature and 0.15 mV for mature connections (0.01 mV bootstrap standard deviation) with ratio 0.75 compatible with the EPSP amplitude ratio (Fig. 1*G*) in immature and mature A1R-activated cases.

### A1R activation and mature synaptic dynamics

**Figure 3:**
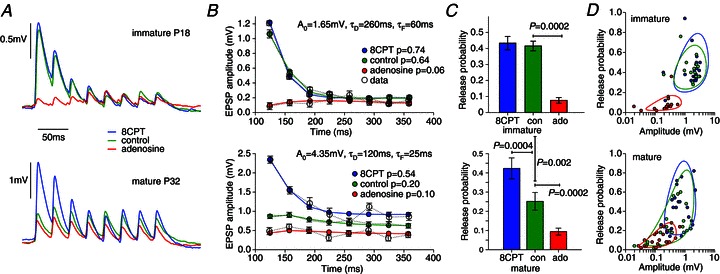
A1R activation underlies the developmental shift from depression to facilitation at L5PC synapses *A*, typical sweep-averaged EPSPs for immature and mature connections in response to eight 30 Hz presynaptic APs under conditions of: A1R block (blue), endogenous A1R activation in control (green), strong A1R activation by applied adenosine (red). Blocking A1Rs causes mature connections to revert to immature synaptic depression. *B*, average EPSP amplitude fits to a synaptic dynamics model for the same connections. Maximal amplitude *A*_0_, and the depression τ_D_ and facilitation τ_F_ time constant fits were common to all pharmacological conditions with release probability *p* optimized for each condition. *C*, mean release probabilities across fitted connections. *D*, scatter plot of release probability *versus* amplitude for individual connections. The overlap of the different distributions follows the same pattern seen for the CV (Fig. 2*C*) and failure rates (Fig. 2*F*) with no evidence seen for the developmental pruning of a distinct population of high-release immature synapses.

To examine how developmental changes in A1R activation affect short-term synaptic plasticity we measured the postsynaptic response to trains of presynaptic APs under the three conditions (Fig. 3*A*). A typical postsynaptic response to 30 Hz APs for an immature connection under control conditions showed synaptic depression (paired-pulse ratio (PPR) of 0.5) with the A1R-blocked condition almost coincident (PPR of 0.4), whereas in applied adenosine the reduced amplitudes exhibited facilitation (PPR of 1.5). For a typical mature connection the response in control conditions showed weak facilitation (PPR of 1.1) as expected (Reyes & Sakmann, 1999). The response was marginally more facilitating in adenosine (PPR of 1.2) but on A1R block significantly increased its initial amplitude and reverted to immature synaptic depression (PPR of 0.7); hence, the facilitation seen in this mature connection is due to A1R activation.

Changes in short-term plasticity can be more globally quantified by fitting to a model of synaptic dynamics (Tsodyks *et al.* 1998) with parameters for the depression time constant τ_D_ and facilitation time constant τ_F_, release probability *p* and maximum EPSP strength *A*_0_ such that *pA*_0_ is the average first EPSP amplitude (see Methods). Parameters used for the example waveform fits are given in Fig. 3*B* and, in general, we found that simultaneous changes in amplitude and synaptic dynamics were well captured (root mean square errors: 0.10 mV for juvenile; 0.05 mV for mature synapses) by only changing release probability between different pharmacological conditions. However, it should be noted that the facilitation and depression time constants were not well constrained for the relatively flat EPSP amplitude profiles in adenosine. We therefore cannot rule out that there might be changes in these parameters under A1R activation, measurable at stimulation frequencies outside the range used here. We applied this fitting procedure, using a variety of different stimulation patterns (20–60 Hz), to measure the probability of release across connections with mean values for *p* of 0.43(0.04), 0.42(0.03) and 0.08(0.02) for immature connections (*N*= 18) and 0.43(0.05), 0.25(0.04) and 0.10(0.02) for mature connections (*N*= 21) for 8CPT, control and adenosine conditions, respectively (Fig. 3*C*). No significant difference was seen between the A1R-blocked state and control for immature connections, whereas for mature connections control release probability was midway between and significantly different from the A1R antagonized (*P*= 0.0004) and agonized (*P*= 0.0002) conditions. Comparing across developmental stages, there was no significant difference in the release probability between the A1R-blocked condition providing further evidence for the reduction in EPSP amplitudes comprising a presynaptic A1R-dependent component (reduced release probability) and postsynaptic A1R-independent component (product of quantal amplitude and release-site number).

A scatter plot of release probability *versus* amplitude for all connections (immature, *N*= 18; mature *N*= 21) under each condition was made to investigate if distinct groups of high-release-probability synapses could be discerned in immature connections that were absent in the mature neocortex (Fig. 3*D*). To give a visual indication of the clustering, bivariate Gaussians were again fitted to logarithms of the data. The structure of distributions was similar to that for the CV-*versus*-amplitude and failure data (Fig. 2*C* and *F*). For immature synapses the distributions for control and 8CPT were almost coincident whereas for mature connections the control distribution showed considerably more spread. These data do not support the hypothesis that a distinct population of high-release-probability synapses are pruned over development but rather are consistent with greater and more heterogeneous activation of A1Rs in control in the mature neocortex.

### A1R activation by endogenous adenosine

To estimate the developmental increase in A1R activation we measured adenosine concentration–response curves using a method that accounts for endogenous adenosine (see Methods). The relative amplitude (*E*−*E*_cpa_)/(*E*_8cpt_−*E*_cpa_) was measured for unitary EPSPs of amplitude *E* measured in 2 μm 8CPT, control, and 10, 30 and 100 μm adenosine, and in 5 μm of the high-affinity A1R agonist CPA (Fig. 4*A*). The average IC_50_ for immature (*N*= 6) and mature (*N*= 4) synapses were coincidently both 15 μm with SEM ranges of 9–23 μm and 6–40 μm, respectively. In three out of six of the immature connections no endogenous A1R activation was measurable, and the average relative control amplitude was 87% that of the A1R-blocked maximum: this is a mean endogenous adenosine concentration of 2(0.8–4) μm. However, all mature synapses had measurable endogenous adenosine concentrations with mean 23(10–50) μm which led to an average relative control amplitude of 40% of the maximal A1R-blocked amplitude. It should be emphasized that these concentrations, like nearly all quoted in the literature, are bath-applied equivalents. Adenosine is rapidly removed from the extracellular space by a number of mechanisms (Haas & Selbach, 2000; Dunwiddie & Masino, 2001) and so the concentration at the synapses is less (by a factor of ∼20, Dunwiddie & Diao, 1994) than that in the bath.

### Heterogeneity of A1R activation

The concentration–response curves (Fig. 4*A*) show that EPSP suppression in 100 μm adenosine is close to the maximally A1R-activated CPA condition. We can therefore estimate the activation fraction of A1Rs in control using the larger set of data for which EPSPs in 8CPT, control and 100 μm adenosine only were measured. Figure 4*B* plots the percentage A1R activation (*E*_8cpt_−*E*_con_)/(*E*_8cpt_−*E*_ado_) *versus* control amplitude *E*_con_ for immature (*N*= 33) and mature (*N*= 18) connections (with a ±20% leeway to allow for measurement uncertainty). The average A1R activations in control conditions were significantly different (*P* < 10^−5^) for the immature (14%) and mature neocortex (64%). The general trend (exponential fit Fig. 4*B*) is that weak synapses have higher A1R activation and therefore endogenous levels of adenosine determine, to a significant extent, the mature EPSP amplitude under control conditions. There is also considerable variability in A1R activation (also seen in Figs 2*C* and *F*, and 3*D*) for both immature and mature connections. Dotted lines are used to associate data points measured from the same slice (usually reciprocal connections) from which it can be seen that the spread in A1R activation is significant even within the same neocortex. To quantify this observation we compared the median spread in A1R activation for synapses from the same neocortex with the average spread between synapses across all data sets (see Methods) within a particular developmental stage (Fig. 4*C*). For example, the 20% (*N*= 13) average spread for the same-cortex immature connections was calculated from the mean height of all dotted lines in Fig. 4*B* that join the same-cortex immature data points and the average spread across all immature data of 18% (*N*= 528) is the mean height between permutations of all immature data points. No significant difference was seen in the spread of activation in immature connections within the same neocortex *versus* the spread in all immature connections, with similar results seen for mature connections: 34% (*N*= 5) median spread within the same slice and 34% (*N*= 153) median spread across all data. These results provide evidence that heterogeneity in A1R activation is not due to variability between individuals with distinct, spatially uniform neocortical adenosine concentrations, but rather that adenosine concentration is a local variable that varies significantly from synapse to synapse, particularly within the mature neocortex.

## Discussion

We provided evidence that increased activation of presynaptic A1Rs mediates the developmental reduction in release probability that partly underlies the reduced unitary amplitude and fully underlies the increase in variability, decrease in reliability and change in synaptic dynamics from depression to facilitation previously observed at L5PC synapses (Reyes & Sakmann, 1999; Frick *et al.* 2007; Feldmeyer & Radnikow, 2009; Etherington & Williams, 2011). We also identified an A1R-insensitive postsynaptic component that appears principally due to decreased effective quantal amplitude (potentially from longer mature dendrites) but could also be due to a decreased number of contacts between L5PCs. Given that the developmental change of decreased release probability is not specific to L5PCs, but has been seen throughout the neocortex, hippocampus, cerebellum and striatum (Feldmeyer & Radnikow, 2009), the A1R-dependent mechanism identified here could generalize to other connections in the mature nervous system.

Double-exponential fits to a use-dependent blockade of NMDA-receptor-mediated currents (Cheetham & Fox, 2010; Gonzalez-Burgos *et al.* 2008) provided evidence for two populations of synapses with high and low release probability, with the former population diminishing during development. The same authors also suggested their data might be interpreted as reflecting shifts in more continuous distributions and our results favour this latter intepretation. We found no evidence for the pruning of a distinct population of high release probability connections but instead saw a spreading-out from a tightly distributed population of immature connections with little endogenous A1R activation to a significantly more heterogeneous mature population with all degrees of A1R activation represented (Figs 2*C* and *F*, and 3*D*).

A number of mechanisms might contribute to increased A1R activation in the mature neocortex. The sensitivity of A1Rs and the G-protein-coupled transduction pathways linking them to suppression of neurotransmitter release may alter over development. However, our data showed an almost identical response of immature and mature synapses to A1R activation (identical ratios of A1R-blocked to -agonized data, Figs 1*G*, 2*A* and *D*, and 3*C*; and the similar IC_50_ values for the immature and mature concentration–response curves, Fig. 4*A*) suggesting little change in A1R affinity or amplification of the transduction mechanism. Alternatively, endogenous extracellular adenosine might increase over development leading to higher A1R activation, and an increased adenosine tone has indeed been seen in the aged nervous system (Sebastião *et al.* 2001; Rex *et al.* 2005). Though diffusive, adenosine is efficiently cleared from the extracellular space (Dunwiddie & Masino, 2001) and its concentration in tissue will most likely be heterogeneous and dependent on: the local distribution of sources, such as breakdown of astrocytically released ATP, direct neuronal release from presynaptic terminals or postsynaptic cells as a retrograde messenger (Latini & Pedata, 2001; Wall & Dale, 2008; Klyuch *et al.* 2011; Lovatt *et al.* 2012); and removal mechanisms, such as transporter-mediated uptake or enzymatic breakdown (Dunwiddie & Masino, 2001). The heterogeneity seen in the present study (Figs 2*C* and *F*, 3*D* and 4*B*) in the levels of A1R activation at mature connections supports this picture. Given the heterogeneous nature of A1R activation by localized and maintained levels of adenosine, and also that this mechanism is one of the key determinants of synaptic strength in the mature somatosensory L5PC network, emplacement of adenosine sources and sinks near synapses with existing A1Rs could constitute a novel form of long-term plasticity in the adult neocortex.

## Abbreviations

A1R: adenosine A_1_ receptor
AP: action potential
CPA: *N*^6^-cyclopentyladenosine
8CPT: 8-cyclopentyltheophylline
CV: coefficient of variation
L5PC: layer 5 pyramidal cell
PPR: paired-pulse ratio
